# Threats to bioethical principles in medical practice in Brazil: new medical ethics code period

**DOI:** 10.1590/1414-431X20176988

**Published:** 2018-03-15

**Authors:** G.C.L. Gracindo, J.H. da Silva Gallo, R. Nunes

**Affiliations:** 1 Faculdade de Medicina, Universidade do Porto, Porto, Portugal; 2 Conselho Federal de Medicina, Brasília, DF, Brasil

**Keywords:** Bioethics, Ethics codes, Medical ethics, Threats

## Abstract

We aimed to outline the profile of medical professionals in Brazil who have violated the deontological norms set forth in the ethics code of the profession, and whose cases were judged by the higher tribunal for medical ethics between 2010 and 2016. This survey was conducted using a database formed from professional ethics cases extracted from the plenary of the medical ethics tribunal of the Federal Council of Medicine. These were disciplinary ethics cases that were judged at appeal level between 2010 and 2016. Most of these professionals were male (88.5%) and their mean age was 59.9 years (SD=11.62) on the date of judgment of their appeals, ranging from 28 to 95 years. Most of them were based in the southeastern region of Brazil (50.89%). Articles 1 and 18 of the medical ethics code were the rules most frequently violated. The sentence given most often was the cancellation of their professional license (37.6%) and the acts most often sentenced involved malpractice, imprudence, and negligence (18.49%). It is acknowledged that concern for the principles of bioethics was present in the appeal decisions made by the plenary of the medical ethics tribunal of the Federal Council of Medicine.

## Introduction

The comprehension of moral issues within medical activity has been discussed since the days of Hippocrates. It involves elements that demarcate medical activities and signal the need for doctors to be aware of their responsibility towards patients and repair possible damages as a result of misconduct. Bioethical principles have become the fundamental basis for the development of professional practice and have come to dictate a particular way of defining and managing the values involved in the relationships between healthcare professionals and their patients (1). With time and consequent evolution of scientific and legal knowledge, views on the medical profession and its consequences for patients' lives have also changed (2).

These issues have been taken up especially by the field of ethics in professional medical practice. However, although applied ethics using the principles of bioethics has become a reality, violation of these deontological rules within the medical practice in Brazil has grown (3,4). Therefore, this subject is extremely important for the medical profession itself and especially for the Brazilian population. This is the consequence for the innumerous investigations and professional ethics cases opened within regional medical councils to investigate ethical violations both nationally (3,4) and internationally (5).

Several factors may be related to the increased numbers of cases. Among these, the following can be highlighted: a) greater awareness among the population regarding their rights; b) deterioration of working conditions, particularly in the public sector; c) influence of the media; d) deterioration of the quality of the doctor-patient relationship; and e) insufficient training for doctors at undergraduate and postgraduate levels, especially from the bioethics point of view (4).

However, there are only few studies investigating the profile of doctors who violate these principles within the Brazilian context. Studies with this objective were conducted previously (3,4), but all included a part of the medical population, generally from one specific regional medical council. Thus, investigations including the entirety of Brazilian territory are scarce. For example, Bitencourt et al. (3) analyzed ethical cases relating to medical errors, but only of doctors registered with the Regional Medical Council of Bahia. These authors observed that the majority of the cases involved male doctors, with a mean age around 40 years, and related to obstetrics and gynecology (23.2%) and general surgery (8.8%).

The ethical code of the medical profession includes 25 fundamental principles of the practice of medicine, 10 norms of medical rights, 118 deontological rules of ethics and four general provisions. Only transgressions of the deontological rules subject the professional to penalties provided by law. It needs to be highlighted that both the current (6) and previous codes of medical ethics (7) contain systematic guidelines that determine appropriate medical practice, and that were defined based on the principles of bioethics. Thus, decisions made by the Federal Council of Medicine (regulatory board of physicians in Brazil) in cases of professional ethics have corroborated that medical practice should be in line with these norms. Failure to do so would be a flagrant violation (6,7).

Infractions of this nature are judged by the Upper Tribunal for Medical Ethics of the Federal Council of Medicine. The tribunal is composed of 27 counsellors who are elected at assemblies of doctors in each state and one full member and his/her respective alternate representing the Brazilian Medical Association. This tribunal is the highest body within the Federal Council of Medicine and it has the power to judge appeals submitted based on decisions in professional ethics cases mostly made by the chambers of the Federal Council of Medicine, or based on decisions to revoke a professional license made by regional medical councils. Revocation of the license is the most severe penalty that is applied to doctors who have seriously violated the deontological norms and this always has to be imposed by the Federal Council of Medicine. License revocation is definitive and cannot be reversed.

Based on the above, the present investigation had the general aim of outlining the profile of Brazilian physicians who violated the deontological norms that are set forth in the professional ethics code and were judged by the Upper Tribunal for Medical Ethics of the Federal Council of Medicine between 2010 and 2016. In addition to highlighting the theoretical importance of the established bioethical principles, the present study also sought, at a practical level, to deepen the knowledge about the judicial actions of the Federal Council of Medicine plenary court and, especially, its analysis on these appeals, thus establishing a descriptive portrayal of the data.

## Material and Methods

### Sample

The Federal Council of Medicine had 429,353 medical professionals registered in 2016. Considering that professionals can have more than one registration (the legislation requires that doctors are registered in each federal state in which they practice), the overall population for this study was the sum of active primary (429,353) and secondary registrations (36,821), thus totaling 466,174 registrations. The medical population consisted of 241,588 men (56.27%) and 187,765 women (43.73%), while the number of registrations was distributed between 265,425 men (61.82%) and 200,749 women (46.75%). Currently, the largest proportion of registered professionals are located in the state of São Paulo (129,326; 27.74%), followed by the state of Rio de Janeiro (64,818; 13.9%) and Minas Gerais (49,455; 10.61%), including both primary and secondary registrations.

Between 2010 and 2016, 2,616 appeals were filed with the Federal Council of Medicine, and a further 414 had already been filed before the study period and were awaiting judgment. Thus, the total number was 3,030 appeals, of which 224 were judged by the plenary body and 2449, by the chambers of the Federal Council of Medicine. Thus, 357 appeals were still awaiting judgment at the end of this period.

The sample for the present study consisted of 206 appeals and 19 referrals, thus totaling 224 appeals from doctors and/or patients that were submitted for judgment by the plenary body of the Upper Tribunal for Medical Ethics of the Federal Council of Medicine, taking into consideration cases that were judged between April 13, 2010, and August 3, 2016. These data were obtained in 2016 from the Federal Council of Medicine database and through consulting the decisions handed down by the plenary body of the Upper Tribunal for Medical Ethics. Three databases were used in the present study: cases (224), doctors sued (191) and cases/penalties (146).

### Procedures

This survey used the database of professional ethics cases judged at appeal level by the plenary body obtained from the Federal Council of Medicine. In addition, demographic distribution data were also gathered. Cases were named appeals if filed by the applicant, and referrals if even without any manifestation of the parties are judged ad referendum by the Plenary of the Federal Council of Medicine, as the penalty is license revocation, under the terms of the law (8).

Cases that were opened at the first court level by regional medical councils before 2010 but received final judgment by the plenary body of the Federal Council of Medicine during this period were taken into consideration. The starting date for this study (April 13, 2010) was the date on which it was enforced the new medical ethics code.

The results are presented as frequencies in the following order: age group at the time of the judgment; gender; federal state; medical specialty; article of the medical ethics code of 2009 that was more often violated; penalty applied; and the year of sentence.

It should be mentioned that the judgments of the chambers of the Upper Tribunal for Medical Ethics of the Federal Council of Medicine were not used. Rather, only the appeals to the plenary body were used. The results from the latter take precedence over judgments in professional ethics cases handed down as majority decisions by the chambers of the Federal Council of Medicine, or over judgments to revoke professional licenses handed down by regional medical councils. The powers of the Upper Tribunal for Medical Ethics are regulated through resolutions by the Federal Council of Medicine and the law that created this body.

### Data analysis

Data were analyzed using the Excel and SPSS (version 21, USA) statistical packages. Descriptive statistics such as the mean, standard deviation and frequency counts were used.

## Results

One hundred and ninety-one doctors were cited in appeals. Sometimes the same doctor appeared in two or more different cases, and this information was taken into consideration in the results. Most of the professionals were male (88.5%) and their mean age was 59.9 years (standard deviation, SD=11.62), ranging from 28 to 95 years. Table 1 presents the distribution according to gender and age in a more detailed manner.

**Table 1.** Age groups of doctors with a lawsuit according to gender.

Age group	Men		Women		Total	
	n	%	n	%	n	%
25 to 35 years	1	0.6	1	4.5	2	1
36 to 45 years	18	10.7	6	27.3	24	12.6
46 to 55 years	38	22.5	4	18.2	42	2
56 to 65 years	52	30.8	10	45.5	62	32.5
66 to 75 years	47	27.8	1	4.5	48	25.1
76 years and over	13	7.7	-	-	13	6.8
Total	169	88.48	22	11.51	191	100

Data are reported as number and percent of n=191 doctors.

Table 1 shows that most of these doctors were in the age group from 56 to 65 years at the time when their cases were judged, corresponding to 0.067% of the male doctors and 0.011% of the female doctors of the total number of doctors registered during the study period.

The distribution of the cases according to regions of Brazil and the federal states is shown in Table 2. The majority of professionals cited in cases were located in the Southeastern region (50.89%), followed by the Central-Western region (20.09%), Southern region (11.16%), Northeastern region (9.82%), and Northern region (8.03%). The distribution according to the states was as follows: São Paulo (28.1%), Rio de Janeiro (12%), Minas Gerais (9.4%), Goiás (7.1%), and Paraná, Pernambuco, Santa Catarina, and Mato Grosso (4.4% each). Furthermore, the proportion of the total number of doctors of southeastern region was 0.05% for men, and 0.01% for women.

**Table 2.** Medical lawsuits per Brazilian region and federal state.

Federal region and state	Men		Women		Total	
	n	%	n	%	n	%
Central-Western region	42	93.33	3	6.66	45	20.08
Federal District	7	3.5	–	–	7	3.1
Goiás	15	7.5	1	4.3	16	7.1
Mato Grosso	11	5.5	1	4.3	12	5.4
Mato Grosso do Sul	9	4.5	1	4.3	10	4.5
Northeastern region	19	86.36	3	13.63	22	9.82
Bahia	4	2.0	1	4.3	5	2.2
Ceará	4	2.0	–	–	4	1.8
Pernambuco	8	4.0	2	8.67	10	4.5
Rio Grande do Norte	3	1.5	–	–	3	1.3
Northern region	17	94.44	1	5.55	18	8.03
Acre	1	0.5	–	–	1	0.4
Amapá	1	0.5	–	–	1	0.4
Amazonas	5	2.5	1	4.3	6	2.7
Pará	5	2.5	–	–	5	2.2
Rondônia	2	0.9	–	–	2	0.9
Tocantins	3	1.5	–	–	3	1.3
Southeastern region	100	87.71	14	12.28	114	50.89
Espírito Santo	2	1.0	1	4.3	3	1.3
Minas Gerais	19	9.5	2	8.7	21	9.4
Rio de Janeiro	24	11.9	3	13.0	27	12.1
São Paulo	55	27.4	8	34.8	63	28.1
Southern region	23	92.0	2	8.0	25	11.16
Paraná	9	4.5	1	4.3	10	4.5
Rio Grande do Sul	5	2.5	–	–	5	2.2
Santa Catarina	9	4.5	1	4.3	10	4.5
Total	201	89.73	23	10.26	224	100

Data are reported as number and percent of n=224 cases.

The specialties for which there were penalties are detailed in Table 3. It should be noted that the data include three cases of preventive interdictions, but these are not considered penalties. The articles of the ethical code most often infringed were 18, 1, 30, 14, and 40. The infraction of article 18 is "Disobeying or disrespecting the judgments and resolutions of the Federal and Regional Councils of Medicine", which is a general regulation. The second and third most infringed articles were "Causing harm to patients through action or omission that could be characterized as malpractice, imprudence or negligence" and "Using the profession to corrupt customs or commit or favor crime". It is also important to mention that article 14 was the most severely punished, since it had the largest number of license cancellations. The category "Others" corresponds to articles that were infringed less than ten times. The table correlating the articles of the medical ethics codes of 1988 and 2009 was used in the judgments in this study. In summary, Table 4 presents the total frequencies and numbers by penalty.

**Table 3.** Specialty or field of activity of doctors with infractions.

Specialty	Men		Women		Total	
	n	%	n	%	n	%
Obstetrics and Gynecology	27	20.1	1	8.3	28	19.1
Internal Medicine	17	12.7	2	16.7	19	13.0
Plastic Surgery	15	11.2	3	25.0	18	12.3
Healthcare Administration	15	11.2	1	8.3	16	11.0
Endocrinology	10	7.5	3	25.0	13	8.9
Psychiatry	6	4.5	–	–	6	4.1
Ophthalmology	6	4.5	–	–	6	4.1
Forensic Medicine and Medical Examiner	6	4.5	–	–	6	4.1
Cardiology	5	3.7	1	8.3	6	4.1
Pediatrics	3	2.2	1	8.3	4	2.7
Urology	3	2.2			3	2.1
Radiology and Imaging diagnostics	2	1.5	1	8.3	3	2.1
Geriatrics and Gerontology	3	2.2	–	–	3	2.1
Anesthesiology	3	2.2	–	–	3	2.1
Rheumatology	2	1.5	–	–	2	1.4
Orthopedics and Traumatology	2	1.5	–	–	2	1.4
Vascular Surgery	2	1.5	–	–	2	1.4
Digestive Tract Surgery	2	1.5	–	–	2	1.4
Hematology and Hemotherapy		1.5	–	–	2	1.4
General Surgery	1	0.7	–	–	1	0.7
Head and Neck Surgery	1	0.7	–	–	1	0.7
Cancerology	1	0.7	–	–	1	0.7
Total	134	100	12	100	146	100

Data are reported as number and percent of n=146.

**Table 4.** Articles of the 2009 medical code of ethics that were infringed.

Article	Total	License cancellation	Publicly censured	Suspended for 30 days	Confidential censure	Confidential warning
18	38 (8.19%)	22 (6.83%)	8 (11.76%)	1 (2.56%)	7 (23.33%)	0 (0.0%)
1	34 (7.33%)	20 (6.21%)	7 (10.29%)	6 (15.38%)	1 (3.33%)	0 (0.0%)
14	27 (5.82%)	25 (7.76%)	1 (1.47%)	1 (2.56%)	0 (0.0%)	0 (0.0%)
30	26 (5.60%)	22 (6.83%)	2 (2.94%)	2 (5.13%)	0 (0.0%)	0 (0.0%)
40	18 (3.88%)	15 (4.66%)	2 (2.94%)	0 (0.0%)	1 (3.33%)	0 (0.0%)
32	17 (3.66%)	9 (2.80%)	5 (7.35%)	3 (7.69%)	0 (0.0%)	0 (0.0%)
38	17 (3.66%)	16 (4.97%)	1 (1.47%)	0 (0.0%)	0 (0.0%)	0 (0.0%)
112	16 (3.45%)	12 (3.73%)	3 (4.41%)	1 (2.56%)	0 (0.0%)	0 (0.0%)
111	15 (3.23%)	10 (3.11%)	2 (2.94%)	1 (2.56%)	2 (6.67%)	0 (0.0%)
51	14 (3.02%)	4 (1.24%)	5 (7.35%)	0 (0.0%)	5 (16.67%)	0 (0.0%)
68	14 (3.02%)	9 (2.80%)	2 (2.94%)	3 (7.69%)	0 (0.0%)	0 (0.0%)
17	12 (2.59%)	9 (2.80%)	1 (1.47%)	0 (0.0%)	2 (6.67%)	0 (0.0%)
21	11 (2.37%)	9 (2.80%)	1 (1.47%)	1 (2.56%)	0 (0.0%)	0 (0.0%)
15	10 (2.16%)	9 (2.80%)	0 (0.0%)	1 (2.56%)	0 (0.0%)	0 (0.0%)
31	10 (2.16%)	9(2.80%)	0 (0.0%)	1 (2.56%)	0 (0.0%)	0 (0.0%)
102	10 (2.16%)	8 (2.48%)	0 (0.0%)	1 (2.56%)	1 (3.33%)	0 (0.0%)
Others*	175 (37.32%)	114 (35.40%)	28 (41.18%)	17 (43.59%)	11 (36.67%)	5 (100.0%)

Data are reported as number and percent of n=146 cases with penalty. *Articles with n<10 violations.

Regarding court decisions, the most frequently made in relation to the appeals were license cancellation (37.6%); absolution (15.7%); public censure in an official publication (11.7%); suspension of professional practice for up to 30 days (7.6%); and confidential censure (5.3%). A significant number of appeals (21.42%) were judged not to have merit and were described as annulled, extinguished, revised, etc. There were low frequencies of revision, partial interdiction, total interdiction and revocation decisions (1.78%) (Table 5).

**Table 5.** Decisions of the Federal Council of Medicine by gender.

Decision	Men		Women		Total	
	n	%	n	%	n	%
License cancellation	78	38.8	5	21.7	83	37.1
Absolved	26	12.9	7	30.4	33	14.7
Publicly censured	23	11.4	2	8.7	25	11.2
Suspended for 30 days	15	7.5	2	8.7	17	7.6
Unknown	19	9.5	1	4.3	20	8.9
Accepted	8	4.0	–	–	8	3.6
Revision	3	1.5	–	–	3	1.3
Judgment annulled	3	1.5	1	4.3	4	1.8
Confidential censure	10	5.0	2	8.7	12	5.4
Confidential warning	4	2.0	1	4.3	5	2.2
Extinguished	7	3.5	2	8.7	9	4.0
Total interdiction	2	1.0	–	–	2	0.9
Partial interdiction	1	0.5	–	–	1	0.4
Revoked interdiction	1	0.5	–	–	1	0.4
Total or definitive suspension	1	0.5	–	–	1	0.4
Total	201	100	23	100	224	100

Data are reported as frequency and percent.

Data are reported as number and percent of n=224.

The data of Table 6 show that the acts most often punished were malpractice, imprudence and negligence (18.49%), followed by medical advertising (10.27%) and disrespect of the patient's modesty (10.27%).

**Table 6.** Actions most often punished.

Acts	Frequency	Percent
Malpractice/imprudence/negligence	27	18.49
Advertising	19	13.01
Disrespect for patient's modesty	15	10.27
Abortion and committing a crime	9	6.16
Unfair competition	9	6.16
Disrespect for patient's modesty and committing a crime	9	6.16
Unrecognized treatment	9	6.16
False testimony	6	4.10
Exploitation of medical work	6	4.10
False testimony and committing a crime	3	2.05
Irregular death certification	2	1.36
Improper charging of fees	2	1.36
Committing a crime	2	1.36
Exaggeration of diagnosis or prognosis	2	1.36
Interaction between pharmacy and medicine	2	1.36
Advertising and interaction between pharmacy and medicine	2	1.36
Others	23	15.75
Total	146	100

Data are reported as frequency and percent.

## Discussion

The present study had the main objective of outlining the profile of doctors who violated the deontological norms set forth in the professional code of ethics between 2010 and 2016. The results show that most appeals heard by the plenary body of the Upper Tribunal for Medical Ethics of the Federal Council of Medicine involved male professionals and in the age group from 56 to 65 years. These results are concordant with the findings from previous studies (3,4,9,10), which showed similar profiles.

It needs to be noted that the number of male doctors registered was greater, and the number of registrations at the time of the infraction is relevant. However, this situation has been changing, given that the number of active female registrations in 2016 is significantly greater. Increasingly more women, and more women than men, are now graduating as doctors, which indicates that a "feminization" of the profession is taking place. Therefore, this debate should be deepened in the new future, given that this population might have different professional practice characteristics, choices of workplace, and specialization preferences than found in the present study (10).

The States of São Paulo and Rio de Janeiro were the states with the largest numbers of appeals submitted for judgment by the plenary body. In addition to having the largest number of registered doctors in Brazil, another reason for having more cases might be a greater rigor displayed by the regional councils in these states. However, the data gathered could not confirm this hypothesis. Moreover, these states are in third and second place, respectively, in relation to the ratio of doctors per 1000 inhabitants (2.7 for São Paulo and 3.75 for Rio de Janeiro), and only behind the Federal District, which has 4.28 doctors per 1000 inhabitants (9,10).

The actions of malpractice, imprudence or negligence resulting from poor or adverse results from medical practice due to action or omission by the doctor were the most often punished. This indicates that classical medical errors in which the doctor is answerable because of inappropriate conduct have been punished with greater rigor. These results are in line with studies from Brazil (3,4) and from other countries (5), which have placed malpractice, imprudence or negligence as the main reasons for professional misconduct among doctors.

On the other hand, unethical medical advertising is also unacceptable. This infraction generally consists of presenting patients with promises of cure through miraculous treatments and medications, usually without scientific basis or against evidence-based medicine, thus putting the population's health at risk. However, a considerable proportion of these doctors are penalized for acts that are more related to character and honesty than to technical training, thus proving that dishonest behavior is considered irremediable and beyond the educational effect of mild penalties (11).

Our data indicate that infractions were not committed by specialists, i.e., doctors holding specialist titles, but rather by general doctors practicing that specialty. Obstetrics and gynecology, internal medicine, plastic surgery and healthcare administration are highlighted in this regard. The first three are among the specialties most sought by patients, within both the public healthcare system and private care (12,13), which might explain the high numbers. However, healthcare administration, which is not a specialty but a field of activity, is a matter of concern. Failure of healthcare administration doctors to adhere to ethical standards can affect the whole organization including patients, employees, peers and all healthcare professionals both directly and indirectly (14,15). A specific code of ethics for healthcare managers would be of great value.

Even though the data analyzed came from appeals judged between 2010 and 2016, most of the infractions were committed before this period. Therefore, as future directions, a study seeking to overcome this limitation, taking the present study as a basis for comparison, is recommended.

In conclusion, a concern for bioethics principles is present in appeal decisions submitted to the plenary body of the Upper Tribunal for Medical Ethics of the Federal Council of Medicine. The results found in this research show that physicians who violate the Brazilian legislation (6) within their professional practice, deliberately or unwittingly, careless of their patients' wellbeing, and/or not respecting patient’s autonomy, are being punished by the Brazilian Federal Council of Medicine.
